# Personality predicts self-rated health: considering age differences

**DOI:** 10.3389/fpsyg.2023.1143077

**Published:** 2023-05-02

**Authors:** Weixi Kang

**Affiliations:** Imperial College London, London, United Kingdom

**Keywords:** personality, Big Five, self-rated health, SRH, age

## Abstract

Self-rated health (SRH) refers to the subjective evaluation of one’s own health. Big Five personality traits including Neuroticism, Agreeableness, Openness, Conscientiousness, and Extraversion have been consistently found as significant predictors of SRH. In addition, SRH declines with age, and personality traits change with age. Thus, it is reasonable to speculate that age might moderate the associations between personality traits and SRH. The current study analyzed data from 33,256 participants with a mean age of 45.78 years old and 55.92% females. The current study found that age significantly moderates the associations between Agreeableness, Openness, and Conscientiousness and SRH after controlling for demographic covariates. The current study implies that personality traits relate to SRH differently at different ages. Thus, studies regarding the associations between personality traits and SRH must take the interactions between age and personality traits into account.

## 1. Introduction

Self-rated health (SRH) refers to the subjective evaluation of one’s own health. Moreover, there is a substantial amount of evidence regarding the predictive validity of this single-item measurement of health. For instance, poor SRH is associated with higher risks of chronic disease, steeper cognitive decline, higher risks of dementia, and higher mortality risk across diverse groups (DeSalvo et al., 2006; Montlahuc et al., 2011; Latham and Peek, 2013; Bendayan et al., 2017). Thus, it is of importance to identify factors that contribute to SRH (Jylhä, 2009) given these associations. According to current conceptualizations and knowledge (see Jylhä, 2009), SRH is a construct that is influenced by various factors, from genetic (Harris et al., 2017) to environmental (Meyer et al., 2014).

The Big Five is one of the most used inventories that measure personality traits, which include Neuroticism, Agreeableness, Openness, Conscientiousness, and Extraversion. This relationship between Big Five personality traits and health can be found in a wide range of health outcomes, including biological and functional markers (Stephan et al., 2018a,b), mental health (Hakulinen et al., 2015a; Kang, 2023), risks of disease such as Alzheimer’s disease (Terracciano et al., 2014) and general mortality (Graham et al., 2017).

Besides objective health, personality is also closely related to SRH. For instance, Neuroticism is the tendency of experiencing negative emotions, which has been consistently associated with poor SRH in cross-sectional (Quinn et al., 1999; Goodwin and Engstrom, 2002; Chapman et al., 2006; Löckenhoff et al., 2012; Turiano et al., 2012; Kööts–Ausmees et al., 2016; Stephan et al., 2020) and longitudinal studies (Löckenhoff et al., 2012; Mund and Neyer, 2016; Stephan et al., 2020). Agreeableness is the tendency of being altruistic and trusting, which has been found to be associated with worse SRH in some studies (Turiano et al., 2012), better SRH in others (Goodwin and Engstrom, 2002), and irrelevant in still others (Löckenhoff et al., 2012). Openness refers to the tendency of being curious and unconventional. While some studies have reported that Openness is positively related to SRH (Goodwin and Engstrom, 2002; Löckenhoff et al., 2012; Stephan et al., 2020), others did not find such an association (Turiano et al., 2012; Kööts–Ausmees et al., 2016; Mund and Neyer, 2016; Stephan et al., 2020). Conscientiousness refers to the tendency of being organized and self-disciplined, which is related to better SRH (Goodwin and Engstrom, 2002; Löckenhoff et al., 2012; Turiano et al., 2012; Stephan et al., 2020; Kitayama and Park, 2021). Finally, Extraversion refers to the tendency of being energetic and sociable, which is associated with better SRH (Goodwin and Engstrom, 2002; Löckenhoff et al., 2012; Turiano et al., 2012; Stephan et al., 2020).

Age is also a consistent predictor of SRH given objective health decreases with age. For instance, cardiovascular disease is more prevalent at older ages compared to younger counterparts (Rieker et al., 2010). There are also studies suggesting that health conditions are the most critical for forming the subjective health evaluation change across age, independent of gender (Read and Gorman, 2010). Indeed, studies have found that SRH declines with age (e.g., Andersen et al., 2007; Zajacova et al., 2017). In addition, although being largely stable, Big Five personality traits also vary with age (e.g., Roberts et al., 2006; Allemand et al., 2007; Donnellan and Lucas, 2009; Soto et al., 2011; Harris et al., 2016).

Taken together, this evidence seems to suggest that personality traits and age are closely associated with SRH. Thus, one would hypothesize that age moderates the associations between personality traits and SRH. As one gets older, they tend to have worse objective health and SRH, which makes them unable to be influenced by certain personality traits. The aim of the current study is to test how age might moderate the associations between Big Five personality traits and SRH.

## 2. Materials and methods

### 2.1. Data

The current study extracted data from Wave 3 (collected between 2010 and 2011), Understanding Society: the UK Household Longitudinal Study (UKHLS), which has been collecting annual information *via* surveys from the original sample of UK households since 1991 when it was previously known as The British Household Panel Study (University of Essex, 2022). Data from Wave 3 was used because only data from Wave 3 contains personality measures. After removing participants with missing values, there were 33,256 participants remaining for further analysis with a mean age of 45.78 years old and 55.92% females.

### 2.2. Measures

#### 2.2.1. Personality traits

Personality was measured using the 15-item version of the Big Five Inventory with a Likert scale ranging from 1 (“disagree strongly”) to 5 (“agree strongly”). Scores were reverse-coded when appropriate. The exact set of questions used to ask participants can be found: https://www.understandingsociety.ac.uk/documentation/mainstage/dataset-documentation/term/personality-traits?search_api_views_fulltext=. Studies have revealed that these short Big Five measures have good internal consistency, test-retest reliability, convergent validity, and discriminant validity (Hahn et al., 2012; Soto and John, 2017).

#### 2.2.2. Self-rated health

Participants responded to the question, “In general, would you say your health is…” using a 5-point scale ranging from 1 (excellent) to 5 (very poor). The reliability of this single measurement of subjective health is high (e.g., Lundberg and Manderbacka, 1996). SRH score was reverse coded so now a higher score means better health.

#### 2.2.3. Demographic variables

Demographic controls included age, sex, monthly income, the highest educational qualification, and the present legal marital status. Specifically, age, and monthly income were coded as what they were (continuous), sex was coded as male vs. female, the highest educational qualification was coded as below college vs. college, and the present legal marital status was coded as single vs. married vs. divorced/separated/widowed.

### 2.3. Analysis

A hierarchical regression was used to analyze how age might moderate the link between Big Five personality traits and SRH. Specifically, demographic variables including age, sex, monthly income, the highest educational qualification, and the present legal marital status and personality traits including Neuroticism, Agreeableness, Openness, Conscientiousness, and Extraversion with age by personality traits interactions were taken into the hierarchical regression models as predictors with SRH health. Then participants were put into three groups based on their age including young (mean age −1 SD), mean-age, and older (mean age +1 SD) groups. Three multiple regressions were then conducted for each age group by taking demographics and personality traits as predictors and SRH as the predicted variable. All analyses were conducted on MATLAB 2018a.

## 3. Results

**FIGURE 1:**
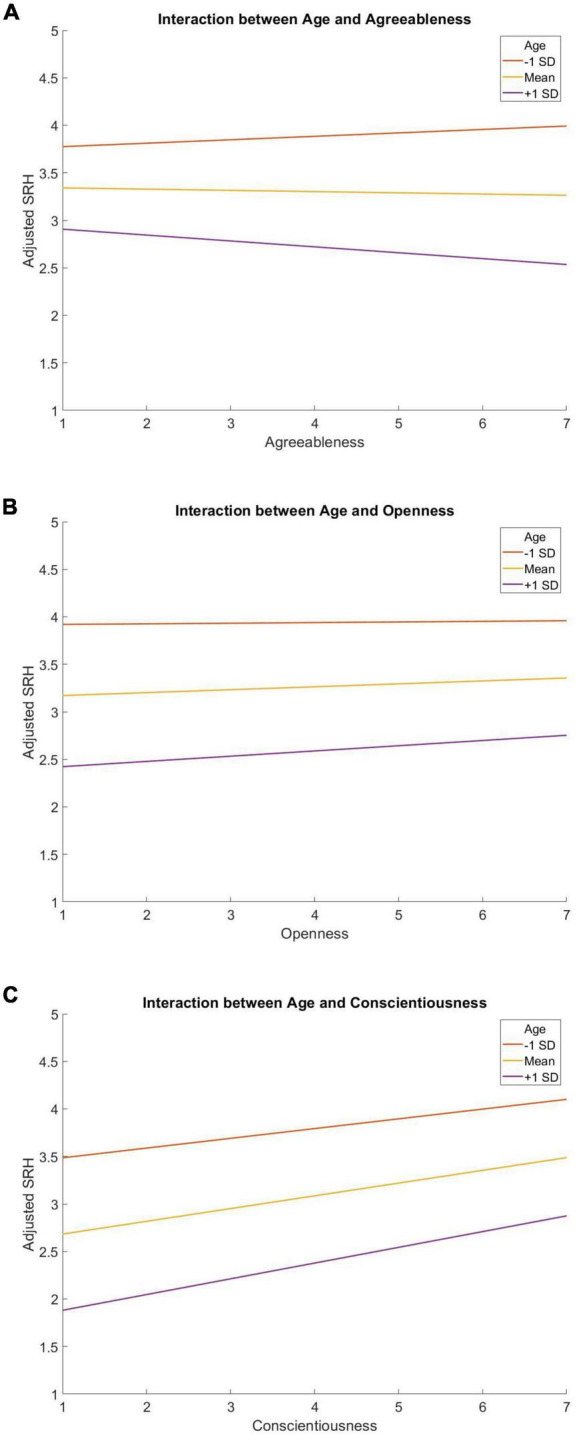
The moderating role of age on the associations between Agreeableness **(A)**, Openness **(B)**, Conscientiousness **(C)**, and SRH.

Descriptive statistics can be found in Table 1. There was a significant interaction of age by Agreeableness (*b* = −0.001, *p* < 0.001, 95% CI [−0.002, −0.001]), Openness (*b* = 0.001, *p* < 0.001, 95% CI [0.001, 0.001]), and Conscientiousness (*b* = 0.001, *p* < 0.001, 95% CI [0.001, 0.001]) interactions after controlling for demographic covariates (Table 2 and Figure 1). One reviewer suggested to code age as categorical (i.e., mean age −1 SD, mean age, mean age +1 SD) rather than continuous before running the same hierarchical regression. However, results were similar (Table 3). Specifically, Neuroticism (*b* = −0.12, *p* < 0.001, 95% CI [−0.13, −0.10]) was negatively related to SRH whereas Agreeableness (*b* = 0.05, *p* < 0.001, 95% CI [0.03, 0.08]), Conscientiousness (*b* = 0.12, *p* < 0.001, 95% CI [0.10, 0.14]) and Extraversion (*b* = 0.03, *p* < 0.01, 95% CI [0.01, 0.05]) were positively related to SRH among young people (mean age −1 SD). However, among mean age adults, Neuroticism (*b* = −0.16, *p* < 0.001, 95% CI [−0.17, −0.15]) was negatively related to SRH whereas Openness (*b* = 0.02, *p* < 0.01, 95% CI [0.01, 0.03]) and Conscientiousness (*b* = 0.14, *p* < 0.001, 95% CI [0.13, 0.15]) were positively related to SRH. Finally, Neuroticism (*b* = −0.14, *p* < 0.001, 95% CI [−0.15, −0.12]) was negatively related to SRH whereas Openness (*b* = 0.05, *p* < 0.001, 95% CI [0.03, 0.07]) and Conscientiousness (*b* = 0.12, *p* < 0.001, 95% CI [0.10, 0.14]) were positively related to SRH in older adults (Table 4).

**TABLE 1:** Descriptive statistics of demographic characteristics, personality traits, and SRH.

Variables	Mean	SD
Age	45.78	17.95
Monthly income	1,363.96	1,363.86
Neuroticism	3.57	1.44
Agreeableness	5.63	1.05
Openness	4.57	1.31
Conscientiousness	5.46	1.12
Extraversion	4.60	1.30
SRH	3.47	1.10
	***N***	**%**
**Sex**		
Male	14,659	44.08
Female	18,597	55.92
**Highest educational qualification**		
Below college	23,198	69.76
College	10,058	30.24
**Legal marital status**		
Single	16,343	49.20
Married	16,874	50.80
Divorced/separated/widowed	5,548	16.68

**TABLE 2:** The regression coefficient (*b*) for demographics, personality traits, and age (continuous) by personality traits interactions with the total explained variances (*R*^2^).

Variables	*b*	95% CI
Age	−0.01***	[−0.02, −0.01]
Sex	0.08***	[0.05, 0.10]
Monthly income	0.00***	[0.00, 0.00]
Highest educational qualification	0.24***	[0.22, 0.27]
Marital status	−0.05***	[−0.07, −0.03]
Neuroticism	−0.15***	[−0.17, −0.13]
Agreeableness	0.05***	[0.03, 0.08]
Openness	0.00	[−0.03, 0.02]
Conscientiousness	0.09***	[0.06, 0.12]
Extraversion	0.03*	[0.01, 0.06]
Age × Neuroticism	0.00	[0.00, 0.00]
Age × Agreeableness	−0.001***	[−0.002, −0.001]
Age × Openness	0.001*	[0.001, 0.001]
Age × Conscientiousness	0.001**	[0.001, 0.001]
Age × Extraversion	0.00	[0.00, 0.00]
*R* ^2^	0.151	

*p < 0.05, **p < 0.01, and ***p < 0.001.

**TABLE 3:** The regression coefficient (*b*) for demographics, personality traits, and age (categorical) by personality traits interactions with the total explained variances (*R*^2^).

Variables	*b*	95% CI
Age	−0.36***	[−0.50, −0.22]
Sex	0.09***	[0.06, 0.11]
Monthly income	0.00***	[0.00, 0.00]
Highest educational qualification	0.28***	[0.25, 0.30]
Marital status	−0.13***	[−0.15, −0.11]
Neuroticism	−0.16***	[−0.18, −0.13]
Agreeableness	0.06**	[0.02, 0.10]
Openness	−0.01	[−0.04, 0.02]
Conscientiousness	0.07***	[0.03, 0.11]
Extraversion	0.04**	[0.01, 0.07]
Age × Neuroticism	0.01	[−0.01, 0.02]
Age × Agreeableness	−0.03**	[−0.05, −0.01]
Age × Openness	0.02*	[0.003, 0.03]
Age × Conscientiousness	0.03**	[0.01, 0.04]
Age × Extraversion	−0.01	[−0.03, 0.00]
*R* ^2^	0.135	

*p < 0.05, **p < 0.01, and ***p < 0.001.

**TABLE 4:** The regression coefficient (*b*) for demographics and personality traits with the total explained variances (*R*^2^) for young (mean age −1 SD), mean age, and older people (mean age +1 SD).

	Young (mean age–1 SD)	Mean age	Older (mean age +1 SD)
Age	-0.03***	-0.02***	-0.02***
Sex	-0.09**	0.09***	0.19***
Monthly income	0.00	0.00***	0.00***
Highest educational qualification	0.20***	0.29***	0.23***
Marital status	-0.04	-0.03*	-0.09***
Neuroticism	-0.12***	-0.16***	-0.14***
Agreeableness	0.05***	-0.01	-0.03
Openness	0.00	0.02**	0.05***
Conscientiousness	0.12***	0.14***	0.12***
Extraversion	0.03**	0.01	0.00
*R* ^2^	0.086	0.139	0.098

*p < 0.05, **p < 0.01, and ***p < 0.001.

As requested by one reviewer, participants were put into three groups including young (<40), middle-aged (>39 and <60), and older people (>59). Similarly, analysis steps were taken but this time age was coded as categorical (i.e., young, middle-aged, and older) rather than continuous before running the same hierarchical regression and multiple regressions. Results were still pretty similar (Tables 5, 6).

**TABLE 5:** The regression coefficient (*b*) for demographics, personality traits, and age (categorical) by personality traits interactions with the total explained variances (*R*^2^).

Variables	*b*	95% CI
Age	−0.32***	[−0.43, −0.21]
Sex	0.08***	[0.06, 0.10]
Monthly income	0.00***	[0.00, 0.00]
Highest educational qualification	0.24***	[0.21, 0.26]
Marital status	−0.12***	[−0.14, −0.10]
Neuroticism	−0.16***	[−0.18, −0.14]
Agreeableness	0.05***	[0.02, 0.08]
Openness	0.005	[−0.02, 0.03]
Conscientiousness	0.06***	[0.03, 0.09]
Extraversion	0.04**	[0.01, 0.06]
Age × Neuroticism	0.01	[0.00, 0.02]
Age × Agreeableness	−0.03***	[−0.04, −0.02]
Age × Openness	0.01*	[0.003, 0.02]
Age × Conscientiousness	0.03***	[0.02, 0.04]
Age × Extraversion	−0.01*	[−0.02, 0.00]
*R* ^2^	0.14	

*p < 0.05, **p < 0.01, and ***p < 0.001.

**TABLE 6:** The regression coefficient (*b*) for demographics and personality traits with the total explained variances (*R*^2^) for young (<40), middle-aged (>39 and <60), and older people (>59).

	Young	Mean age	Older
Age	−0.02*** [−0.03, −0.02]	−0.02*** [−0.02, −0.01]	−0.02*** [−0.02, −0.01]
Sex	−0.05* [−0.08, −0.01]	0.11*** [0.07, 0.15]	0.22*** [0.17, 0.27]
Monthly income	0.00*** [0.00, 0.00]	0.00*** [0.00, 0.00]	0.00*** [0.00, 0.00]
Highest educational qualification	0.22*** [0.18, 0.26]	0.30*** [0.26, 0.35]	0.25*** [0.19, 0.32]
Marital status	0.03 [0.00, 0.07]	−0.05** [−0.08, −0.02]	−0.12*** [−0.16, −0.07]
Neuroticism	−0.13*** [−0.14, −0.12]	−0.18*** [−0.19, −0.17]	−0.14*** [−0.16, −0.12]
Agreeableness	0.04*** [0.02, 0.05]	−0.02 [−0.04, 0.00]	−0.03* [−0.05, 0.00]
Openness	0.00 [−0.01, 0.02]	0.02* [0.00, 0.04]	0.04*** [0.03, 0.06]
Conscientiousness	0.12*** [0.10, 0.13]	0.15*** [0.13, 0.17]	0.13*** [0.11, 0.15]
Extraversion	0.03*** [0.01, 0.04]	0.00 [−0.01, 0.02]	0.00 [−0.02, 0.02]
*R* ^2^	0.095	0.138	0.104

*p < 0.05, **p < 0.01, and ***p < 0.001.

## 4. Discussion

The aim of the current study was to test how age may moderate the link between personality traits and SRH in a large representative sample from the United Kingdom. Results from the current study showed that age significantly moderates the associations between Agreeableness, Openness, and Conscientiousness and SRH after controlling for demographic covariates. Specifically, Neuroticism was negatively related to SRH whereas Agreeableness, Conscientiousness, and Extraversion were positively related to SRH among young people. However, among mean-age and older adults, Neuroticism was negatively related to SRH whereas Openness and Conscientiousness were positively related to SRH.

Neuroticism was consistently negatively associated with SRH in all age groups, which seems to be largely consistent with previous studies. A direct explanation for the negative association between Neuroticism and SRH is that neurotic individuals tend to have worse health, which can then be reflected in SRH. Moreover, Neuroticism is also associated with slower walking speed (e.g., Stephan et al., 2018a,b) and biological dysfunctions (Sutin et al., 2019). Moreover, Neuroticism is a consistent predictor of poor health outcomes including major depression (Hakulinen et al., 2015a), dementia (Terracciano et al., 2014), and chronic respiratory diseases (Terracciano et al., 2017). Moreover, processes underlying Neuroticism can also explain poor SRH. For instance, individuals with neuroticism may tend to perceive the word negatively so they rated their health as actually worse than their objective health (Sutin and Terracciano, 2016). Finally, the negative association between Neuroticism and SRH can be explained by shared genetics. For instance, Harris et al. (2017) found that scores for Neuroticism were negatively related to SRH.

Conscientiousness was positively related to SRH in all age groups. Conscientiousness tends to be positively associated with health-promoting behaviors such as more physical activities (Sutin et al., 2016; Kroencke et al., 2019) but negatively related to health-risk behaviors such as alcohol use (Hakulinen et al., 2015b) and smoking (Luchetti et al., 2018), which can then have an effect on SRH. In addition, Conscientiousness is also negatively associated with the risk of chronic diseases (Weston et al., 2015) such as obesity (Jokela et al., 2013). Moreover, SRH is negatively related to depressive symptoms over time (Hakulinen et al., 2015a), which may result in better SRH. Conscientiousness is also positively related to lung function, grip strength, and walking speed (e.g., Stephan et al., 2018a,b), which may contribute to better SRH. There may be underlying psychophysiological mechanisms that explain the positive association between Conscientiousness and SRH. For instance, Conscientiousness is positively associated with metabolic, inflammatory, and cardiovascular markers (Luchetti et al., 2014; Sutin et al., 2018) and higher cardiorespiratory fitness (Terracciano et al., 2013). These better biomedical profiles may lead to better SRH. Moreover, there was a significant moderation effect of age on the association between Conscientiousness and SRH, with a moderate association in young people, the strongest association in mean-age adults, and the weakest association in older adults, which may be explained by the fact mean-age adults have the highest levels of Conscientiousness comparing to younger or older people (e.g., Donnellan and Lucas, 2009).

The current study found that Agreeableness is positively related to SRH in young people but not in young or older adults. The positive association between Agreeableness and SRH seems to be consistent with previous studies (Strickhouser et al., 2017). The positive association between Agreeableness and SRH may be explained by the fact that Agreeableness is associated with more physical activities (Artese et al., 2017). However, the relationship between Agreeableness and health in older adults seems to be mixed in the literature, while the current study did not find that Agreeableness relates to SRH, others found that Agreeableness is negatively connected to health (e.g., Turiano et al., 2012). Older adults may not enjoy the benefits of Agreeableness such as more physical activities due to their declining health, which may accompany with functional limitations.

Finally, Openness was only positively related to SRH in middle-aged and older adults. Indeed, recent studies have found that Openness is associated with more physical activities (Sutin et al., 2016), better physical functioning (Stephan et al., 2018a,b), and a lower inflammation rate (Luchetti et al., 2014), which may, in turn, result in better SRH. However, Openness did not relate to SRH in young participants, which may also in turn reflect on the mixed findings regarding Openness and SRH in the literature. This difference can be explained by the fact that younger people are generally healthy so the benefit of Openness to health is none. Moreover, during emerging adulthood, people with high Openness may be more vulnerable to risky behaviors that are detrimental to health such as illegal drug use (Kang, 2022), which may even relate to negative SRH. Moreover, the positive relationship between Openness and SRH was slightly large in older adults, which may be explained by the fact people with high Openness may perceive better social cohesion (Larsen et al., 2020), and social cohesion is positively related to SRH in older adults (Chumbler and Leech, 2013).

Despite the strengths of the current study including a large sample size and well-controlled socioeconomic characteristics, there are some limitations of the current study as well. First, the current study relied on a cross-sectional design, which cannot establish causality. Second, the current study was based on self-reported measures, which cannot avoid self-reporting bias. Specifically, since both personality and health are subjective evaluations, which mean that they share the method. Thus, they are not entirely independent entities and the observed relationships between them can be produced by the artifact of the method. Finally, the current study focused on participants from the United Kingdom, which may make it generate to other cultures/countries (e.g., Kitayama and Park, 2021). Future studies should use longitudinal approaches, more objective measures, and samples from multiple cultures/countries.

Taken together, the current study found that age significantly moderates the associations between Agreeableness, Openness, and Conscientiousness and SRH after controlling for demographic covariates. Specifically, Neuroticism was negatively related to SRH whereas Agreeableness, Conscientiousness, and Extraversion were positively related to SRH among young people. However, among mean-age and older adults, Neuroticism was negatively related to SRH whereas Openness and Conscientiousness were positively related to SRH. The current study implies that personality traits relate to SRH differently at different ages. Thus, studies regarding the associations between personality traits and SRH must take the interactions between age and personality traits into account.

## Data availability statement

Publicly available datasets were analyzed in this study. This data can be found here: https://www.understandingsociety.ac.uk.

## Ethics statement

The studies involving human participants were reviewed and approved by the University of Essex. Written informed consent to participate in this study was provided by the participants’ legal guardian/next of kin.

## Author contributions

The author confirms being the sole contributor of this work and has approved it for publication.

## References

[B1] Allemand M. Zimprich D. Hertzog C. (2007). Cross-sectional age differences and longitudinal age changes of personality in middle adulthood and old age. *J. Pers.* 75, 323–358. 17359241 10.1111/j.1467-6494.2006.00441.x

[B2] Andersen F. K. Christensen K. Frederiksen H. (2007). Self-rated health and age: A cross-sectional and longitudinal study of 11,000 Danes aged 45—102. *Scand. J. Public Health* 35 164–171. 10.1080/14034940600975674 17454920

[B3] Artese A. Ehley D. Sutin A. R. Terracciano A. (2017). Personality and actigraphy-measured physical activity in older adults. *Psychol. Aging* 32:131. 10.1037/pag0000158 PMC5369413 28287783

[B4] Bendayan R. Piccinin A. M. Hofer S. M. Muniz G. (2017). Are changes in self-rated health associated with memory decline in older adults? *J. Aging Health* 29 1410–1423. 27481931 10.1177/0898264316661830

[B5] Chapman B. P. Duberstein P. R. Sörensen S. Lyness J. M. (2006). Personality and perceived health in older adults: The five factor model in primary care. *J. Gerontol. Series B Psychol. Sci. Soc. Sci.* 61 362–365. 10.1093/geronb/61.6.p362 17114306

[B6] Chumbler N. R. Leech T. (2013). “The impact of neighborhood cohesion on older individuals’ self-rated health status,” in *Social determinants, health disparities and linkages to health and health care* (Bingley: Emerald Group Publishing Limited), 31, 41–55. 10.1177/0164027512475096 24860203 PMC4030643

[B7] DeSalvo K. B. Bloser N. Reynolds K. He J. Muntner P. (2006). Mortality prediction with a single general self-rated health question. *J. Gen. Intern. Med.* 21 267–275. 16336622 10.1111/j.1525-1497.2005.00291.x PMC1828094

[B8] Donnellan M. B. Lucas R. E. (2009). Age differences in the Big Five across the life span: Evidence from two national samples. Psychol. *Aging* 23, 558–566. 10.1037/0882-7974.23.3.558 PMC2562318 18808245

[B9] Goodwin R. Engstrom G. (2002). Personality and the perception of health in the general population. *Psychol. Med.* 32 325–332. 11866326 10.1017/s0033291701005104

[B10] Graham E. K. Rutsohn J. P. Turiano N. A. Bendayan R. Batterham P. J. Gerstorf D. (2017). Personality predicts mortality risk: An integrative data analysis of 15 international longitudinal studies. *J. Res. Pers.* 70 174–186. 29230075 10.1016/j.jrp.2017.07.005 PMC5722274

[B11] Hahn E. Gottschling J. Spinath F. M. (2012). Short measurements of personality–validity and reliability of the GSOEP Big Five Inventory (BFI-S). *J. Res. Pers.* 46 355–359.

[B12] Hakulinen C. Elovainio M. Pulkki-Råback L. Virtanen M. Kivimäki M. Jokela M. (2015a). Personality and depressive symptoms: Individual participant meta-analysis of 10 cohort studies. *Depress. Anxiety* 32 461–470. 26014798 10.1002/da.22376 PMC4605994

[B13] Hakulinen C. Hintsanen M. Munafò M. R. Virtanen M. Kivimäki M. Batty G. D. (2015b). Personality and smoking: Individual-participant meta-analysis of nine cohort studies. *Addiction* 110 1844–1852. 26227786 10.1111/add.13079 PMC4609271

[B14] Harris M. A. Brett C. E. Johnson W. Deary I. J. (2016). Personality stability from age 14 to age 77 years. *Psychol. Aging* 31:862. 10.1037/pag0000133 PMC5144810 27929341

[B15] Harris S. E. Hagenaars S. P. Davies G. David Hill W. Liewald D. C. Ritchie S. J. (2017). Molecular genetic contributions to self-rated health. *Int. J. Epidemiol.* 46 994–1009. 27864402 10.1093/ije/dyw219 PMC5837683

[B16] Jokela M. Hintsanen M. Hakulinen C. Batty G. D. Nabi H. Singh-Manoux A. (2013). Association of personality with the development and persistence of obesity: A meta-analysis based on individual–participant data. *Obes. Rev.* 14 315–323. 10.1111/obr.12007 23176713 PMC3717171

[B17] Jylhä M. (2009). What is self-rated health and why does it predict mortality? Towards a unified conceptual model. *Soc. Sci. Med.* 69 307–316. 19520474 10.1016/j.socscimed.2009.05.013

[B18] Kang W. (2022). Big Five personality traits predict illegal drug use in young people. *Acta Psychol.* 231:103794. 10.1016/j.actpsy.2022.103794 36368191

[B19] Kang W. (2023). The associations between personality traits and mental health in people with and without asthma. *J. Affect. Disord.* 333:102–106. 10.1016/j.jad.2023.04.022 37075823

[B20] Kitayama S. Park J. (2021). Is conscientiousness always associated with better health? A US–Japan cross-cultural examination of biological health risk. *Pers. Soc. Psychol. Bull.* 47 486–498. 10.1177/0146167220929824 32552349 PMC7746573

[B21] Kööts–Ausmees L. Schmidt M. Esko T. Metspalu A. Allik J. Realo A. (2016). The role of the five–factor personality traits in general self–rated health. *Eur. J. Pers.* 30 492–504. 21299558

[B22] Kroencke L. Harari G. M. Katana M. Gosling S. D. (2019). Personality trait predictors and mental well-being correlates of exercise frequency across the academic semester. *Soc. Sci. Med.* 236:112400. 10.1016/j.socscimed.2019.112400 31336217

[B23] Larsen M. M. Esenaliev D. Brück T. Boehnke K. (2020). The connection between social cohesion and personality: A multilevel study in the Kyrgyz Republic. *Int. J. Psychol.* 55 42–51. 10.1002/ijop.12551 30485424 PMC7027491

[B24] Latham K. Peek C. W. (2013). Self-rated health and morbidity onset among late midlife US adults. *J. Gerontol. Series B Psychol. Sci. Soc. Sci.* 68 107–116. 10.1093/geronb/gbs104 PMC3605944 23197340

[B25] Löckenhoff C. E. Terracciano A. Ferrucci L. Costa P. T. Jr. (2012). Five-factor personality traits and age trajectories of self-rated health: The role of question framing. *J. Pers.* 80 375–401. 21299558 10.1111/j.1467-6494.2011.00724.x PMC3248623

[B26] Luchetti M. Barkley J. M. Stephan Y. Terracciano A. Sutin A. R. (2014). Five-factor model personality traits and inflammatory markers: New data and a meta-analysis. *Psychoneuroendocrinology* 50 181–193. 10.1016/j.psyneuen.2014.08.014 25233337 PMC4544833

[B27] Luchetti M. Sutin A. R. Delitala A. Stephan Y. Fiorillo E. Marongiu M. (2018). Personality traits and facets linked with self-reported alcohol consumption and biomarkers of liver health. *Addict. Behav.* 82 135–141. 10.1016/j.addbeh.2018.02.034 29525559 PMC5890805

[B28] Lundberg O. Manderbacka K. (1996). Assessing reliability of a measure of self-rated health. *Scand. J. Soc. Med.* 24 218–224. 8878376 10.1177/140349489602400314

[B29] Meyer O. L. Castro-Schilo L. Aguilar-Gaxiola S. (2014). Determinants of mental health and self-rated health: A model of socioeconomic status, neighborhood safety, and physical activity. *Am. J. Public Health* 104 1734–1741. 10.2105/AJPH.2014.302003 25033151 PMC4151927

[B30] Montlahuc C. Soumare A. Dufouil C. Berr C. Dartigues J. F. Poncet M. (2011). Self-rated health and risk of incident dementia: A community-based elderly cohort, the 3C study. *Neurology* 77 1457–1464. 10.1212/WNL.0b013e31823303e1 21975209

[B31] Mund M. Neyer F. J. (2016). The winding paths of the lonesome cowboy: Evidence for mutual influences between personality, subjective health, and loneliness. *J. Pers.* 84 646–657. 10.1111/jopy.12188 26112403

[B32] Quinn M. E. Johnson M. A. Poon L. W. Martin P. (1999). Psychosocial correlates of subjective health in sexagenarians, octogenarians, and centenarians. *Issues Ment. Health Nurs.* 20 151–171. 10.1080/016128499248727 10409994

[B33] Read J. N. G. Gorman B. K. (2010). Gender and health inequality. *Annu. Rev. Sociol.* 36 371–386. 10.1146/annurev.soc.012809.102535

[B34] Rieker P. P. Bird C. E. Lang M. E. (2010). “Understanding gender and health: Old patterns, new trends, and future directions,” in *Handbook of medical sociology*, eds Bird C. Conrad P. Fremont A. Timmermans S. (Nashville, TN: Vanderbilt University Press), 52–74. 10.2307/j.ctv16h2n9s.7

[B35] Roberts B. W. Walton K. E. Viechtbauer W. (2006). Patterns of mean-level change in personality traits across the life course: A meta-analysis of longitudinal studies. *Psychol. Bull.* 132, 1–25. 16435954 10.1037/0033-2909.132.1.1

[B36] Soto C. J. John O. P. (2017). Short and extra-short forms of the Big Five Inventory–2: The BFI-2-S and BFI-2-XS. *J. Res. Pers.* 68 69–81. 10.1016/j.jrp.2017.02.004

[B37] Soto C. J. John O. P. Gosling S. D. Potter J. (2011). Age differences in personality traits from 10 to 65: Big Five domains and facets in a large cross-sectional sample. *J. Pers. Soc. Psychol.* 100, 330–348. 21171787 10.1037/a0021717

[B38] Stephan Y. Sutin A. R. Bayard S. Križan Z. Terracciano A. (2018a). Personality and sleep quality: Evidence from four prospective studies. *Health Psychol.* 37:271. 10.1037/hea0000577 29172602 PMC5837948

[B39] Stephan Y. Sutin A. R. Bovier-Lapierre G. Terracciano A. (2018b). Personality and walking speed across adulthood: Prospective evidence from five samples. *Soc. Psychol. Pers. Sci.* 9 773–780. 10.1177/1948550617725152

[B40] Stephan Y. Sutin A. R. Luchetti M. Hognon L. Canada B. Terracciano A. (2020). Personality and self-rated health across eight cohort studies. *Soc. Sci. Med.* 263:113245. 10.1016/j.socscimed.2020.113245 32810694

[B41] Strickhouser J. E. Zell E. Krizan Z. (2017). Does personality predict health and well-being? A metasynthesis. *Health Psychol.* 36:797. 10.1037/hea0000475 28277701

[B42] Sutin A. R. Terracciano A. (2016). Five-factor model personality traits and the objective and subjective experience of body weight. *J. Pers.* 84 102–112. 10.1111/jopy.12143 25329238

[B43] Sutin A. R. Stephan Y. Luchetti M. Artese A. Oshio A. Terracciano A. (2016). The five-factor model of personality and physical inactivity: A meta-analysis of 16 samples. *J. Res. Pers.* 63, 22–28. 29056783 10.1016/j.jrp.2016.05.001 PMC5650243

[B44] Sutin A. R. Stephan Y. Terracciano A. (2018). Facets of conscientiousness and objective markers of health status. *Psychol. Health* 33 1100–1115. 10.1080/08870446.2018.1464165 29718717 PMC6286646

[B45] Sutin A. R. Stephan Y. Terracciano A. (2019). Personality and metabolic dysfunction in young adulthood: A cross-sectional study. *J. Health Psychol.* 24 495–501. 10.1177/1359105316677294 27837153

[B46] Terracciano A. Schrack J. A. Sutin A. R. Chan W. Simonsick E. M. Ferrucci L. (2013). Personality, metabolic rate and aerobic capacity. *PLoS One* 8:e54746. 10.1371/journal.pone.0054746 23372763 PMC3556088

[B47] Terracciano A. Stephan Y. Luchetti M. Gonzalez-Rothi R. Sutin A. R. (2017). Personality and lung function in older adults. *J. Gerontol. Series B Psychol. Sci. Soc. Sci.* 72 913–921. 10.1093/geronb/gbv161 26786321 PMC5926981

[B48] Terracciano A. Sutin A. R. An Y. O’Brien R. J. Ferrucci L. Zonderman A. B. (2014). Personality and risk of Alzheimer’s disease: New data and meta-analysis. *Alzheimers Dement.* 10 179–186. 23706517 10.1016/j.jalz.2013.03.002 PMC3783589

[B49] Turiano N. A. Pitzer L. Armour C. Karlamangla A. Ryff C. D. Mroczek D. K. (2012). Personality trait level and change as predictors of health outcomes: Findings from a national study of Americans (MIDUS). *J. Gerontol. Series B Psychol. Sci. Soc. Sci.* 67 4–12. 10.1093/geronb/gbr072 21765062 PMC3410684

[B50] Weston S. J. Hill P. L. Jackson J. J. (2015). Personality traits predict the onset of disease. *Soc. Psychol. Pers. Sci.* 6 309–317. 10.1177/1948550614553248

[B51] Zajacova A. Huzurbazar S. Todd M. (2017). Gender and the structure of self-rated health across the adult life span. *Soc. Sci. Med.* 187 58–66. 10.1016/j.socscimed.2017.06.019 28654822 PMC5554534

